# Determining a common understanding of interprofessional competencies for pre-registration health professionals in Aotearoa New Zealand: A Delphi study

**DOI:** 10.3389/fmed.2023.1119556

**Published:** 2023-03-24

**Authors:** Patrea Andersen, Patrick Broman, Ema Tokolahi, Jia Rong Yap, Sharon Brownie

**Affiliations:** ^1^Centre for Health and Social Practice, Waikato Institute of Technology–Te Pūkenga, Hamilton, New Zealand; ^2^School of Nursing, Midwifery and Paramedicine, University of the Sunshine Coast, Sippy Downs, QLD, Australia; ^3^School of Nursing, Midwifery and Social Science, Central Queensland University, Norman Gardens, QLD, Australia; ^4^Otago Polytechnic–Te Pūkenga, Dunedin, New Zealand; ^5^School of Health Science, Swinburne University of Technology, Melbourne, VIC, Australia; ^6^School of Medicine and Dentistry, Griffith University, Brisbane, QLD, Australia

**Keywords:** interprofessional education, competencies, assessment, curriculum transformation, Delphi, health workforce education, New Zealand

## Abstract

There is growing awareness that factors such as the growing incidence of co-morbidity and increasing complexity of patient health needs cannot be addressed by health professionals practicing in isolation. Given this, there is an increasing emphasis on preparing students in health-related programs for effective interprofessional practice. Less clear, however, are the specific skills and clinical or learning opportunities necessary for students to develop effectiveness in interprofessional practice. These factors drove a team associated with a tertiary health education provider in Hamilton, New Zealand to transform traditional clinical student experiences in the form of an interprofessional student-assisted clinic. The clinic was intended, in part, to provide students with opportunities to learn and experience interprofessionalism in practice but was hampered by limited information available regarding the specific skill requirements necessary for students in New Zealand to learn in this context. In this Delphi study, we synthesize national expert opinion on student competency indicators necessary for effective interprofessional practice. The resultant set of indicators is presented and opportunities for application and further research discussed. The paper offers guidance to others seeking to innovate health curricula, develop novel service-oriented learning experiences for students, and foster interprofessional practice competence in the future health workforce.

## 1. Introduction

Similar to other developed nations, the growing burden of poor population health in Aotearoa New Zealand has been well publicized (1). The Ministry of Health (MoH) records that over the last 10 years, the estimated rate of diabetes among New Zealanders has increased from 35.7 per 1,000 population in 2012 to 41.5 in 2021. Adult and child obesity are also on the rise, with 1 in 3 adults (34.3%) and 1 in 8 children (12.7%) classified as obese in 2019/2020. MoH further reports that adults and children living in socioeconomically deprived areas were 1.6 times and 2.5 times as likely to be obese as those living in the least deprived areas. The challenge of these health issues, and health disparities requires both an understanding of the principles of non-communicable disease management and control and the role of environmental factors in health and disease (2). Moreover, the increasing complexity of patient care requirements and the incidence of co-morbidity requires health and social service practitioners to collaborate in the delivery of care (3). In other words, to be effective in addressing the needs of the population, health interventions increasingly require the collective efforts of professionals from a very wide array of fields (4).

Within the educational context, this calls for curricula and programs of study which place an emphasis on interprofessional practice, known as interprofessional education (IPE) (5, 6). In one definition, IPE is understood to occur when “students from two or more professions learn about, from and with each other to enable effective collaboration and improve health outcomes” [1 p. 13, (7)]. How IPE is conceptualized and practiced can vary depending on the country and the healthcare setting. Further to this, regulations on interprofessional competencies vary across countries (8, 9). Despite this variability, some commonly described themes and elements include effective communication, mutual respect, teamwork, reflexive practice, leadership and management, and ethical considerations (10–12). While general and didactic, such competencies help to grow healthcare professionals that can share information and ideas, to negotiate and resolve conflicts, and to work effectively as part of multidisciplinary healthcare teams in the management of complex patient care. This has been shown to result in improved healthcare experiences and outcomes and reduced healthcare costs (10, 13, 14).

this background there is an urgent need for academic institutions to respond with opportunities for students to learn and demonstrate competency in an interprofessional context (5, 6) and develop competency assessment tools to accommodate assessment of students practicing in this context (15, 16). To do so, Loura et al. (11) suggest a competency-based approach, aligning interprofessional competency frameworks to learning-outcomes based curricula to ensure proper appropriation of knowledge and implementation of IPE in practice to enable learners to take more responsibility for their own learning and development (17). Grymonpre et al. (18) argue that successful implementation of IPE requires collaborative efforts at macro-, meso-, and micro- levels. For example, they suggest creating partnerships between higher education institutions, government and practice communities (macro-level), developing and revising a strategic IPE plan (meso-level), and adopting common frameworks and language (micro-level) when advancing IPE. Put together, they highlight the importance of creating authentic, experiential IPE for health professionals in training as knowledge cannot be independent of the situations where it is learned and applied (19). However, understaffing and complex workplace issues fueling the lack of clinical placements make offering students opportunities to work with other professions across the sector difficult. These circumstances call for creative curriculum delivery and create an opportunity for a more “personalized learning” model with an emphasis on heutagogical (student-driven) models of education, underpinned by real-world application of knowledge and skills that involve problem and project-based learning (20).

One approach internationally to transforming health curricula and enhancing the development of interprofessional practice has been the introduction of student-assisted or student-led clinics (21–23). One such project, He Kaupapa Oranga Tahi, based in Hamilton, New Zealand, was initiated with the aim of promoting and integrating IPE. The project undertaken by our team is funded by Trust Waikato with these overarching objectives: establish an interprofessional center of clinical training excellence preparing health professionals with a high level of clinical capability, cultural competence, and community insight. In this clinic, Waikato Institute of Technology–Te Pūkenga students from different professions (nursing, physiotherapy, occupational therapy, social work, counseling, exercise physiology, and sports science), working under the supervision of health professionals, undertake community-based clinical experience providing primary health care as a team, strongly networked to community-based health and social service provider partners. The clinic focuses on diabetes, heart disease, and mobility and falls prevention as particular healthcare needs in the local community (1, 22). The intention is for the clinic to provide an opportunity for students to come to an understanding of their professional role and that of other professionals they work with, and how collectively as a team they contribute to health care outcomes (12, 21, 22). The development of this innovative clinical and placement experience prompted consideration of suitable curricula for involved students, particularly around the skills and competencies required to operate effectively in this interprofessional environment.

In a tertiary education context, it is essential that assessment of competency requirements is consistent with the learning outcomes defined in each discipline’s program of study and meet the requirements of each discipline’s regulatory body. Although there is an identified need for more studies investigating behavior-based or competency-based outcomes of interprofessional practice (15, 16, 22), there is no consensus about what these outcomes should include, particularly for pre-registration health professionals in New Zealand. Various tools and frameworks exist internationally (24–30), and the Otago Interprofessional Education Conceptual Model (31) outlines domains of competency relevant to the New Zealand context, consistent with international literature. However, no behavioral or performance indicators were found to complement this model and no known competency tools have been found that assess pre-registration (as opposed to graduate level) interprofessional competencies across health disciplines in New Zealand. To facilitate the development of a competency assessment tool suitable for assessing practice expectations of students working in an interprofessional student-assisted clinic, this enquiry sought to determine expert consensus, from academic staff supporting the learning of students from a range of health disciplines, to the question “What knowledge, skills and behaviors do health discipline experts consider indicators of competent student interdisciplinary practice in New Zealand?’

Detailed examination of this topic in New Zealand is timely, given the health programme unification currently underway as part of the government’s wider Reform of Vocational Education (RoVE) programme. These reforms have included the merger of 16 existing local “Institutes of Technology and Polytechnics” (ITPs), and 9 industry training organizations (ITOs) into a large, unified non-university tertiary education provider–Te Pūkenga–The New Zealand Institute of Skills and Technology (32). Its predecessor organizations delivered or arranged over 2,000 programs to more than 200,000 learners across the country, including health programmes leading to registration in most fields outside of medicine and dentistry. Significant work is currently underway developing single, “unified” programs of study for the newly established entity to deliver across the country in each learning category (32). This unification of multiple programmes of study into single programmes gives further impetus for defining a collective national view of necessary competencies for effective interprofessional practice across disciplines. Understanding student practice expectations and embedding the interprofessional domains and indicators into one framework will set the foundation for genuinely responsive best practices to be cultivated, implemented, and assessed in these unified programmes.

## 2. Materials and methods

### 2.1. Study design

The study followed a Delphi methodology for determining consensus on a question of interest from experts in the field (33). This well-established technique is common in healthcare and nursing fields (34). With an underlying assumption that expert consensus is more valid than individual opinion, it involves two or more rounds of questionnaires amongst a panel of experts. Qualitative/open-ended questions aimed at identifying key factors from experts typically feature in early rounds, while later rounds are primarily quantitative, with experts indicating the extent to which they agree with various identified factors of interest (33, 35). Expert opinions are synthesized after each round and only those on which there is consensus are retained in future rounds, with the panel thus guided toward agreement. The number of rounds used is best determined by the level of consensus shown amongst expert participants.

A modified Delphi methodology was adopted as the aim was to achieve a collective consensus view of subject academics across a wide range of taught health fields/professions and institutions across Te Pūkenga–the New Zealand Institute of Skills and Technology. Unlike other group methods, a Delphi study does not require the researcher or participants to be located in proximity. In addition, it involves the blinding of participants, who work anonymously and independently from one another. This participant blinding reduces the risk of group dynamics influencing outcomes (33).

### 2.2. Participants

For this study, an expert was defined as a teaching staff member of a health or wellbeing-related Te Pūkenga program, with 5 + years of professional experience in their relevant field, and an interest in interprofessional education and practice. As teaching staff, each were both a health professional with an understanding of both their field and a health-related educator with experience teaching, supervising, and/or assessing healthcare students. We sought an expert sample in which each of the varied health-related fields of study currently taught within Te Pūkenga (nursing, medical imaging, paramedicine, counseling, social work, physiotherapy, osteopathy, massage, sport and exercise science, clinical exercise physiology, and occupational therapy) were represented, and with participants from a range of the 16 institutes of technology and polytechnics which at the time of data collection were undertaking a merger into Te Pūkenga–the New Zealand Institute of Skills and Technology. As a vocational (non-university) education provider, Te Pūkenga does not offer higher-level degrees in fields such as medicine or dentistry. In recognition of this fact, some IPE experts in university medical education programmes in New Zealand were also invited to participate.

The study was approved by the Wintec Human Ethics in Research Group (HERG), approval reference WTLR18170522 dated 20 May 2022, as a low-risk application. Potential participants were provided an information sheet and consent form prior to agreeing to participate, and informed consent was provided electronically *via* an initial survey section with agreement required prior to proceeding. Confidentiality in reporting was assured for participants and names were not collected, although some identifying information such as profession, teaching institution, and job title was asked for and is reported here by consent.

Subsequent to ethical approval, potential participant educators for the study were identified by (a) members of the research team; (b) purposive sampling of experienced staff from a list of all health programs offered by Te Pūkenga subsidiaries (identified *via* institutional websites); (c) snowball sampling whereby individuals asked to participate were also invited to provide details of appropriate experts. Sampling was deliberate inasmuch as at least one appropriate expert was sought from each health field taught in the Te Pūkenga network, and from a range of the 16 institutions undertaking the merger. In addition to teaching staff in Te Pūkenga-taught qualifications, several interprofessional education experts (in medicine) from the New Zealand university sector were also invited to participate, in recognition of their specific expertise in this field. The number of expert participants recruited (*n* = 17) aligns to the Delphi panel size of between 15 and 30 participants recommended by De Villiers et al. (36).

### 2.3. Data tool, collection, and analysis

In the absence of consensus in the New Zealand context around interprofessional competencies necessary for students to develop, an initial survey tool was developed comprising of a list of 78 indicators of interprofessional competencies that were collated from a range of sources (25, 26, 37–42). For the purposes of this inquiry, and guided by the literature, these were considered to group naturally into 6 key competency domains: *communication*; *leadership*; *interpersonal relationships and mutual support*; *monitoring and situational awareness*; *student knowledge*; and *student skills.* The initial (first round) survey also included an open-ended question for participants to indicate any competencies/indicators not included in the initial list.

Data collection was *via* three sequential survey rounds undertaken using SurveyMonkey^®^ internet survey software (SurveyMonkey LLC, Portland, OR, USA), with invite links distributed by email. Partially completed surveys were excluded from analysis, on the assumption that partial completion reveals insight into participants’ level of engagement. To maximize rigor, the order of lists within each key category was randomized for each participant in each round. Analysis of resulting data was undertaken using Excel^®^ (Microsoft, Redmond, WA, USA). The study comprised two rating rounds (rounds 1 and 2) where participants rated the relevance of items (interprofessional competency indicators) on a 1–100 scale and a ranking round (round 3) where participants ranked the remaining items’ importance against each other.

In (rating) round 1, consensus agreement was calculated using a combination of mean scores, indicating levels of importance, and quartile deviation, indicating the level of consensus (43). Items receiving a median score of 70 or greater and with an interquartile range of 20 or less were considered to have high consensus importance and proceeded to ranking round 3. In keeping with previous research (44), indicators in round 1 with median scores below 30 (indicating low importance) and with an interquartile range of 20 or less (indicating high consensus) were considered less important by high consensus and excluded from subsequent rounds. Items not meeting either criterion were considered uncertain and re-rated in round 2. In round 2 a median score of 70 or greater was required for items to proceed to round 3. In the final ranking round (round 3), mean rankings were calculated to indicate the collective expert view of the importance of each item (45).

#### 2.3.1. Survey round 1

The first round was undertaken from 28 June to 10 August 2022. Initial invitations were emailed to 43 potential participants. Those of whom who did not complete were also sent a follow-up reminder email. A total of 17 experts completed the first round of the survey. For each indicator, participants were asked to show, on a 1–100 sliding scale, “the extent to which you agree the attribute, knowledge or skill is required for students to learn and demonstrate developing safe interprofessional practice.” The initial round also included a number of questions regarding participants’ general characteristics, including professional role, number of years in profession and their teaching institution.

#### 2.3.2. Survey round 2

The second round was undertaken from August 14 to 10 September 2022. A total of 15 of the 17 participants of the first survey round completed this round, which asked them to reassess and indicate on a 1–100 scale the level of importance they attached to competency indicators retained from the initial list (consensus on high or low importance not having been achieved) and to do the same for additional indicators not included in the initial list but mentioned by one or more participants in the open-ended question of round 1. The similar scores achieved for retained indicators in this round compared to the first survey round suggested that further rating surveying would not result in significant further clarity and, therefore, results from the current and previous round were taken to provide a conclusive list of relevant competencies and indicators in New Zealand context.

#### 2.3.3. Survey round 3

The final survey round was undertaken 10 October to 18 November 2022 and was completed by 12 of the 17 participants who had completed the earlier rounds. This round asked experts to rank, within each of the 6 key competency domains, the 73 important indicators agreed as relevant from earlier rounds, from most to least important. There is consensus within the literature that the number of items individuals can reasonably rank against each other is around 20 (46, 47). Here, the number of indicators retained to be ranked in the 6 key competency domains varied from 7 (in the knowledge competency domain) to 18 (in the leadership and interpersonal relationships and mutual support competency domains). This round was intended for experts to collectively determine the relative importance of each of the indicators, as an important corollary of identifying the indicators themselves.

## 3. Results

### 3.1. Participant characteristics

Table 1 shows participants’ general characteristics, as indicated in the first survey round. Of the 17 expert participants, fifteen represented six of the 16 predecessor Institute of Technology and Polytechnics (ITPs) of Te Pūkenga. They were from the New Zealand university sector and in roles explicitly related to interprofessional health education. Although no New Zealand-based educator in the field of paramedicine was successfully recruited (only one such program of study exists within Te Pūkenga), a New Zealand clinical educator of paramedicine currently associated with an Australian university was approached and agreed to participate.

**TABLE 1:** Expert panel characteristics (*n* = 17).

	*N*	%
**Gender**		
Male	6	35%
Female	11	65%
**Primary teaching programme/area of expertise**		
Nursing	3	23%
Medical imaging	2	12%
Paramedicine	1	6%
Counseling	1	6%
Social work	1	6%
Physiotherapy	1	6%
Osteopathy	1	6%
Massage	1	6%
Sport and exercise science	1	6%
Clinical exercise physiology	1	6%
Occupational therapy	1	6%
Midwifery	1	6%
Interprofessional education (Medicine)	2	12%
**Years of professional experience**		
Under 10 years	1	6%
10–19 years	4	24%
20–29 years	5	29%
30 + years	7	41%
**Rounds completed**		
Round 1 (1–100 scale rating and open- Ended question)	17	100%
Round 2 (1–100 scale rating)	15	88%
Round 3 (ranking)	12	71%

Regarding participants’ working experience, the shortest and longest time participants had been registered/practiced/taught in their professional field was 5 and 42 years, respectively. The average (mean) number of years of working experience was 25.2 years. Response rates are an important consideration for expert consultation, being an indication of the level of enthusiasm and engagement in the research amongst experts (34). Forty-three (43) experts were initially invited to participate, of whom 17 (40%) completed an initial survey. Subsequent recovery rates were relatively high, with 15 (88%) of the 17 initial participants completing the second round and 12 (71%) completing the third.

### 3.2. Rating rounds–interprofessional competency indicators

Consensus agreement was indicated in the first round by a median rating of 70 or greater and with a quartile deviation of 20 or less. Amongst the 17 experts who completed the first round, there was consensus agreement around the relevance or importance of 36 of the 78 interprofessional competency indicators included in the initial survey instrument. Three items in the initial survey were identified by consensus as unimportant (i.e., had a median score of less than 30 and an interquartile range of 20 or less). These were the leadership competency indicator *helps manage co-location*, and the skills competency indicators *education skills* and *counseling.*

There was uncertainty around 39 of the indicators in the primary survey (those having received a median score between 30 and 70 and/or a quartile deviation over 20). These indicators were re-rated in the second round. Also rated in the second round were 8 additional indicators mentioned by 6 participants in the open-ended question included in the first round. These were *avoiding bullying/antisocial behavior (actively anti-bullying/speaking up*) which was characterized in later rounds as an “interpersonal relationships and mutual support” competency; *reflection, active listening skills, self-awareness, cultural safety/competency to work with difference* and *followership–the ability to take direction well* which were characterized as “student skill” competencies, and *understanding interprofessional values and ethics* and having *ability to work to strengths*, which were both characterized as indicators in the “student knowledge” competency domain. An overview of all competencies and indicators included in the study is provided in Table 2.

**TABLE 2:** Overview of interprofessional competencies and indicators.

Potential interprofessional competencies/Indicators	Round 1			Round 2		Final result
	**Median**	**IQR**	**Result**	**Median**	**Result**	
**Communication**						
Communication that facilitates a shared mental model	85	23	?	82	✓	✓
Effective communication with other professions	100	2	✓			✓
Understands and uses share terminology	100	29	?	98	✓	✓
Understands and uses shared documentation	78	23	?	72	✓	✓
Uses standardized clinical handover	80	49	?	68	–	–
Uses closed loop communication or “check back” to verify information	95	20	✓			✓
Engages in case conference/management	90	24	?	89	✓	✓
Engages in debrief	96	26	?	92	✓	✓
Applies conflict resolution techniques where required	100	20	✓			✓
Communicates respectfully with all members of the healthcare team	100	0	✓			✓
Uses only recognized terms and abbreviations when communicating	90	41	?	77	✓	✓
Promotes own disciplinary perspective within the team	87	31	?	90	✓	✓
**Leadership**						
Engages in collaborative leadership	98	30	?	92	✓	✓
Understands role responsibilities	100	2	✓			✓
Understands the team structure	91	17	✓			✓
Orientates the team	80	29	?	78	✓	✓
Articulates clear goals/plan	90	28	?	83	✓	✓
Includes patients and family whānau as part of the team	90	20	✓			✓
Understands responsibility for assigning tasks/Responsibilities to team members	80	27.75	?	82	✓	✓
Understands responsibility for managing/Allocating resources	80	40	?	76	✓	✓
Helps manage co-location	60	21	–	–	–	–
Engages in practice that maximizes activities of the team	90	14	✓			✓
Recognizes need to balance workloads in the team	90	25	?	83	✓	✓
Facilitates information sharing	100	10	✓			✓
Provides timely feedback to healthcare team	80	29	?	79	✓	✓
Monitors quality/efficiency – Reduction in clinical errors	90	31	?	87	✓	✓
Facilitates conflict resolution	90	40	?	92	✓	✓
Focuses on behaviors not personal attributes	92	10	✓			✓
**Interpersonal relationships and mutual support**						
Models effective teamwork	95	10	✓			✓
Develops role awareness	91	15	✓			✓
Facilitates collaboration	99	15	✓			✓
Understands roles of other health professionals	100	0	✓			✓
Respects knowledge and practice of others	100	7	✓			✓
Provides patient-centered care	100	8	✓			✓
Respects other’s culture/beliefs and values	100	2	✓			✓
Facilitates community and foster a climate where it is expected that assistance will be sought/offered	90	19	✓			✓
Protects others from high workload situations	59	30.75	?	63	–	–
Fosters mutual trust	100	7	✓			✓
Employs strategies to support team functioning	100	14	✓			✓
Anticipates support required	80	15	✓			✓
Anticipates other team members’ needs	78	29.25	?	77	✓	✓
Advocates for the patient	99	18	✓			✓
Works in partnership with other healthcare professionals to define/Articulate common goals	100	15	✓			✓
Works collaboratively to improve health outcomes for individuals	99	15	✓			✓
Works collaboratively to improve health outcomes for populations	98	10	✓			✓
Collaborates to facilitate smooth transmission between services	100	14	✓			✓
Avoiding bullying/antisocial behavior (actively anti- bullying/speaking up)	Identified by experts			87	✓	✓
**Monitoring and situational awareness**						
Monitors environmental safety	99	25	?	94	✓	✓
Monitors context/triage acuity	82	28.75	?	77	✓	✓
Prioritizes actions	90	21	?	93	✓	✓
Uses strategies to monitor team performance	70	33	?	85	✓	✓
“Watches each other’s backs”	83	27	?	80	✓	✓
Ensures mistakes/oversights are addressed quickly	99	21	?	95	✓	✓
Complies with policy and procedures	99	15	✓			✓
Monitors fatigue including psychological issues/stress	86	40	?	72	✓	✓
Monitors progress toward achievement of goals	99	20	✓			✓
Monitors status of team’s patient	99	19	✓			✓
Critically evaluates services delivered	90	38	?	77	✓	✓
Collaborates with the interprofessional team to develop policies and guidelines informed by best available evidence	86	24	?	62	–	–
Collaborates with the interprofessional team to monitor and update policies and guidelines informed by best available evidence	81	33.5	?	69	–	–
Collaborates with the health team to generate new knowledge for the betterment of peoples’ lives, communities and wider society	91	25	?	63	–	–
**Student knowledge**						
Develops an accurate/sound knowledge base	99	14	✓			✓
Understands group dynamics	86	21	?	83	✓	✓
Understands environmental culture	85	20	✓			✓
Develops discipline specific knowledge	98	24	?	98	✓	✓
Engages in shared decision-making	95	20	✓			✓
Medication/pharmacology knowledge	80	26	?	67	–	–
Understanding interprofessional values and ethics	Identified by experts			98	✓	✓
Having ability to work to strengths	Identified by experts			88	✓	✓
**Student skills**						
Health assessment	90	20	✓			✓
Group facilitation	72	47.75	?	60	–	–
Education skills	69	20	–	–	–	–
Counseling	60	17.75	–	–	–	–
Negotiation	70	17	✓			✓
Adaptability	90	20	✓			✓
Patient history	99	16	✓			✓
Vital signs	95	35	?	90	✓	✓
Medication administration	60	34.5	?	58	–	–
Communication	90	15	✓			✓
Reflection	Identified by experts			73	✓	✓
Active listening skills	Identified by experts			85	✓	✓
Self-awareness	Identified by experts			74	✓	✓
Cultural safety/Competency to work with difference	Identified by experts			93	✓	✓
Followership – the ability to take direction well	Identified by experts			89	✓	✓

In the second-round participants (*n* = 15) were asked to rate again the importance of the 38 indicators where no consensus had been achieved and the additional 8 indicators identified by experts. In this round, a median rating of 70 (of 100) was required, a somewhat lesser requirement than the first round where the requirement for high consensus agreement (indicated by the interquartile range) was also included. Further rating rounds would increase the response burden on participants and were considered unlikely to offer significant further clarity. Of the 38 indicators retained from the earlier round, 31 met criteria for inclusion in the final list, although the relatively low consensus around these indicators compared to those that achieved consensus agreement (indicated by the low interquartile range) in the first round should be noted. Of the 8 additional indicators added by participants,’ all 8 achieved consensus agreement for inclusion in the final set of indicators, with median ratings of between 78 (for “reflection”) and 98 (for “understanding interprofessional values and ethics”).

Following rounds 1 and 2, consensus agreement was established around the importance of 73 core indicators or skills necessary for students preparing for interprofessional practice. This final set of indicators are grouped within the higher-level competencies, which could also be thought of as competency domains (24) or core competencies (48) of communication (*n* = 11), leadership (*n* = 15), interpersonal relationships and mutual support (*n* = 18), monitoring and situational awareness (*n* = 11), student knowledge (*n* = 7), and student skills (*n* = 11). Of the higher-level competencies, the highest levels of consensus agreement were shown for the indicators characterized in the “Interpersonal relationships and mutual support” domain. Of the 18 indicators included in this domain, 16 achieved consensus for inclusion in the first survey round. This compares with only 2 of 14 indicators in the monitoring and situational awareness domain that achieved consensus in the first round.

### 3.3. Ranking agreed interprofessional competencies

Following the two-round rating and indicator identification exercise of rounds 1 and 2, experts (*n* = 12) ranked the final consensus set of 78 indicators within each of their 6 competency domains. The aim was for panel experts, given their experience and judgment, to determine the relevance and importance of indicators relative to each other, with obvious implications for the structure and focus of future healthcare training programs. The results of this exercise, with mean ranking scores (lower mean scores indicating higher rankings) are provided as Table 3.

**TABLE 3:** Indicators of interprofessional competency (*n* = 73), ranked within competency domains.

Communication	$\bar{x}$	Leadership	$\bar{x}$	Interpersonal relationships and mutual support	$\bar{x}$	Monitoring and situational awareness	$\bar{x}$	Student knowledge	$\bar{x}$	Student skills	$\bar{x}$
Communicates respectfully with all members of the healthcare team	3.0	Understands the team structure	5.5	Models effective teamwork	4.9	Prioritizes actions	3.7	Understands interprofessional values and ethics	1.9	Self-awareness	4.0
Effective communication with other professions	4.0	Includes patients and family whānau as part of the team	6.7	Works in partnership with other healthcare professionals to define/articulate common goals	6.4	Monitors status of team’s patient	4.4	Develops an accurate/+ sound knowledge base	2.3	Cultural safety/Competency to work with difference	4.4
Engages in debrief	5.7	Engages in collaborative leadership	6.8	Works collaboratively to improve health outcomes for individuals	6.9	Monitors progress toward achievement of goals	5.0	Understands group dynamics	2.9	Communication	4.6
Uses closed loop communication or “check back” to verify information	6.3	Understands role responsibilities	7.6	Understands roles of other health professionals	6.9	Complies with policy and procedures	5.1	Understands environmental culture	3.2	Reflection	4.8
Understands and uses shared documentation (=)	6.6	Engages in practice that maximizes activities of the team	8.6	Fosters mutual trust	7.1	Monitors context/Triage acuity	5.3	Ability to work to strengths	5.1	Active listening skills	5.4
Understands and uses shared terminology (=)	6.6	Articulates clear goals/Plan	8.6	Respects knowledge and practice of others	7.3	Uses strategies to monitor team performance	5.8	Engages in shared decision-making	5.3	Adaptability	6.7
Communication that facilitates a shared mental model	7.2	Facilitates information sharing	9.3	Advocates for the patient (=)	7.7	Monitors environmental safety	6.2	Develops discipline specific knowledge	5.4	Health assessment	6.8
Engages in case conference/management	7.3	Recognizes need to balance workloads in the team	9.8	Respects other’s culture/beliefs and values (=)	7.7	Monitors fatigue including psychological issues/Stress	6.3			Followership –ability to take direction well	7.7
Applies conflict resolution techniques where required	7.8	Orientates the team (=)	10.3	Facilitates collaboration (=)	7.7	Ensures mistakes/Oversights are addressed quickly	7.1			Patient history	8.0
Promotes own disciplinary perspective within the team	8.5	Understands responsibility for assigning tasks/Responsibilities to team members (=)	10.3	Provides patient-centered care	8.0	Critically evaluates services delivered	8.1			Group facilitation	8.2
Uses only recognized terms and abbreviations when communicating	8.6	Provides timely feedback to healthcare team	10.9	Works collaboratively to improve health outcomes for populations	8.9	“Watches each other’s backs”	9.2			Negotiation	9.4
		Monitors quality/Efficiency – Reduction in clinical errors	11.6	Avoiding bullying/antisocial behavior (actively anti-bullying/Speaking up)	10.5						
		Focuses on behaviors not personal attributes	11.9	Employs strategies to support team functioning	10.6						
		Understands responsibility for managing/Allocating resources	12.5	Develops role awareness	11.0						
		Facilitates conflict resolution	12.8	Anticipates other team members needs	12.4						
				Anticipates support required	12.6						
				Facilitates community and foster a climate where it is expected that assistance will be sought/Offered	12.6						
				Collaborates to facilitate smooth transmission between services	13.3						

$\bar{x}$ = mean rank (round 3).

Asked to rank-order indicators to establish priorities amongst items, experts placed the greatest priority on *communicates respectfully within all members of the healthcare team* within the “Communication” competency domain, on *understands the team structure* within the “Leadership” domain, *models effective teamwork* within the “Interpersonal relationships and mutual support” domain, *prioritizes actions* within the “Monitoring and situational awareness” domain, *understands interprofessional values and ethics* within the “Student knowledge” domain and *self-awareness* within the “Student skills” domain. The last two were both indicators identified by experts in the first round, not having been included in the original survey instrument.

## 4. Discussion

The aim of this study was to reach expert agreement on the IPE competencies necessary for students, and more specifically students in an New Zealand public health promotion and interdisciplinary context. A modified Delphi study sought the opinions of 17 expert academic staff teaching in health and social service programs in Te Pūkenga–New Zealand Institute of Skills and Technology, the primary national vocational education provider in New Zealand, and in medical training programmes in the local university sector. In two initial rating rounds, experts agreed with a high consensus on the importance of 73 interprofessional competency indicators. Indicators were initially derived from the literature on interprofessional practice/education, with additions nominated by experts. In the third-round, experts ranked the relative importance of indicators within each of the six domains.

At a high level, experts implicitly agreed, *via* their consensus view of the importance of 73 indicators as important for students to demonstrate in interprofessional practice, that competency in this area for New Zealand students cannot be distilled easily into one skillset, statement, or competency. This finding aligns with a consensus view in the literature that interprofessional practice is both complex and multifaceted (49–51). Expert opinion was generally supportive of the indicators obtained from the literature, which may not be surprising given that the experts were highly experienced teaching staff with an interest in the field. They were likely to be familiar with the literature on interprofessional competency and influenced by it. The alignment and agreement shown by local experts to indicators derived from existing literature do indicate the local relevance of global work in IPE, much of which originates in the United States and the United Kingdom (52, 53). As Green and Johnson (54) point out, all health (and education) systems within which future health care workers must be trained to work together exist in a local context.

One finding of note was that while the higher-level indicator *Promotes own disciplinary perspective within the team* was rated by experts as an important skill within the “communication” domain, more discipline-specific knowledges and skills (e.g., *Medication/pharmacology knowledge, Counseling*, and *Vital signs* etc.) were not recognized by the panel as important in an explicitly interprofessional context. While such discipline-specific knowledges are clearly important for relevant disciplines to be educated in and facilitate promoting one’s own disciplinary perspective within a team, it is assumed the panel did not consider competency in these indicators as vital to all members of healthcare teams aspiring to effective interprofessional practice. This highlights how interprofessional learning outcomes are specific and focused and differ from core disciplinary skills. As O’Keefe et al. (27) suggest, “it can be argued that many interprofessional learning activities comprise core disciplinary competencies that are being taught in an interprofessional context rather than addressing specific interprofessional learning competencies *per se*” (p. 463). Educators should differentiate carefully between developing disciplinary skills or identity and developing interprofessional skills.

A number of higher-order competency indicators related to policy development and monitoring were not rated as important for students by the expert panel. For example, *Collaborates with the health team to generate new knowledge for the betterment of peoples’ lives, communities and wider society* and *Collaborates with the interprofessional team to develop policies and guidelines informed by best available evidence* and *Collaborates with the interprofessional team to monitor and update policies and guidelines informed by best available evidence* did not achieve consensus. This may be attributed to the practice-oriented nature of the survey and the fact panelists were asked to rate and rank necessary competencies for *students* of their disciplines. Such higher-order competency indicators as those may not be considered realistic to expect of health or social service students, or indeed novice practitioners, and may have been considered by the experts as important or relevant interprofessional competencies, though for advanced practitioners. There appears to be limited research exploring how interprofessional competencies develop over time, as practitioners progress from novice through to advanced clinicians. Interprofessional competency is not static and will dynamically evolve over a student’s, and later a clinician’s, time in practice. Panelists in the current study were invited to rank indicators as indicative of student level attainment. However, like practitioners’ progress from novice to more advanced levels of practice, there are graded levels of attainment for students too. For example, exposure, engagement, immersion and mastery of competency indicators might develop over a programme, as suggested in the Otago Interprofessional Education Conceptual Model (31). This level of distinction was not sought in the current study and could be beneficial to investigate in future research. For example, investigating which competencies are mastered earlier or later across a range of health programmes, or exploring if the competency indicators for first year nursing students are comparable with the competency indicators for first year occupational therapy students? The list of interprofessional competency indicators in the current study were found to represent a starting point, or baseline level of competency, from which further development in this area can proceed.

Panel experts identified the indicator *understands interprofessional values and ethics* as a clear oversight in the original survey instrument, perhaps not surprisingly given the ubiquity of reference to interprofessional values and ethics in the literature over time (24, 29), though more frequently framed as an overarching competency domain than indicator (24, 55, 56). When noting additional skills or competencies experts may not have differentiated between domain and indicator, and familiarity with the terms may have led to consensus more than questioning at what level interprofessional values and ethics are demonstrated. The degree of consensus agreement and high ranking of this indicator in subsequent rounds illustrates the extent to which experts agree that the explicit teaching of this content was essential. Effective interprofessional practice requires an understanding of differences in values and beliefs between disciplines (57). Exposure to IPE can promote proficiency for students in balancing views and understanding ethical dilemmas from different standpoints (58).

Of note is that the skill of followership arose as a significant indicator. Not originally included in the survey, followership was identified by experts in the first round and its importance reinforced in subsequent rounds. Followership can be defined as “the willingness to cooperate in a coordinated way to accomplish shared goals while engaging in collaborative teamwork” (59) (p. 82). McKimm and Vogan (60) argue that in developing teamworking and leadership skills, “learning how to be an authentic leader as well as a “proactive” follower can lead to more effective interprofessional teamworking and ultimately an improvement in health outcomes” (p. 41). While leadership-oriented research is historically more prominent, this is an increasing focus in healthcare scholarship that addresses followership, including acknowledging the need for health care clinicians to be “flexible in switching between leader and follower roles as appropriate to advance patient care” (61) (p. 3308). The relationship between followers and leaders is interdependent, followership can potentially reduce burnout, and followers can play a significant role in impacting successful outcomes in the work environment (62). Followership is therefore an important skill for students to develop when planning for an agile workforce and executing public health directives (62).

An additional indicator identified by experts in the first round, and reinforced in later rounds, was that of *cultural safety/competency to work with* difference. Cultural safety is a term that originated from nursing practice in New Zealand (63). As this nation becomes increasingly multicultural, and given its significant ethnic disparities in health, cultural competence must be reflected in healthcare practice. Broadly, this requires practitioners to recognize diverse contexts within and between cultural groups, and the impact of their own culture on their professional practice and interaction with clients (64) and how they work with other health professionals. Practitioners should therefore be able to function respectfully and effectively with people from different backgrounds and contribute positively to quality healthcare and achieving health equity. This is a moral and ethical obligation that is also grounded in New Zealand legislation and manifests in regulatory body requirements reinforcing the role of health practitioners in reducing ongoing inequalities in health status and outcomes, particularly for Māori.

Increasing awareness of this dynamic in the local healthcare context likely played a role in the experts in this panel to identify this as necessary for effective interprofessional practice, though it is anticipated this would have arisen as essential for any interprofessional activity in New Zealand, regardless of the domain of focus. New Zealand is far from the only nation to experience health inequities and with recent social movements (such as Black Lives Matter) fore-fronting greater awareness of inequities, notions of cultural safety and responsiveness have attained international traction (65–67). While there is limited recognition of these concepts in explicitly IPE literature, it is possible that this field is just “catching up.” Taken as a whole, findings from this study seem to illustrate an expert belief that public health initiatives need to be interprofessional and culturally responsive by design; it is at this intersection that positive health outcomes can be achieved and sustained (68, 69).

### 4.1. Limitations

Limitations of this study must be noted. As a modified Delphi method, the study relied primarily on a preliminary list of competencies and indicators drawn from the literature. While this approach minimizes response burden and simplifies the approach, and opportunity was provided for participants to add any items not included to the list for subsequent rounds, this approach does raise the prospect that providing a predetermined list may introduce some level of conformity bias (70, 71). In addition, and although the study included multidisciplinary perspectives from health disciplines taught across in a national context, it only included staff from Te Pūkenga–the New Zealand Institute of Skills and Technology and interprofessional education staff from the New Zealand university sector. It cannot claim to be representative: participants were self-selected, and the sample size relatively low. Some fields (such as social work and sport science) were not represented in the latter rounds of the study. It should also be noted that participants and therefore expert perspectives presented here are exclusively those of academic teaching staff from the various health fields, not those currently engaged in health care practice. Whether these perspectives differ, and the implications for IPE and public health, are matters for further research.

## 5. Conclusion

Given interprofessional collaboration is increasingly required for effective public health services and initiatives, there is a clear need to train and assess health and social practice students in these domains. This modified Delphi study identifies a key set of 73 important interprofessional competency indicators for students in New Zealand, as rated and ranked by experts from a range of health and social service programs. Followership was an unexpected competency indicator that was identified by experts in the first round of this study, aligns with emerging literature and is worthy of further investigation. While identification of cultural competence in the New Zealand context was not unexpected there is a need to determine indicators in this area as they relate to interprofessional practice. This will provide direction for educators and ensure learners can be supported in their development as competent, culturally safe follower-leaders.

The high level of engagement from experts in this study confirms there was a need to clarify expectations of students’ performance in interprofessional practice. Further work is indicated, including the development of a structured student assessment tool based on findings and completing necessary validation to ensure its rigor across a range of students and disciplines, learning providers, and placement settings (72). Nevertheless, the agreed domain and indicator framework presented here provides a local starting point for best practice “interprofessionalism” to be cultivated, implemented, and assessed.

## Data availability statement

The original contributions presented in this study are included in the article/supplementary material, further inquiries can be directed to the corresponding author.

## Ethics statement

The studies involving human participants were reviewed and approved by the Wintec Human Ethics in Research Group Approval Ref: WTLR18170522. The patients/participants provided their written informed consent to participate in this study.

## Author contributions

All authors contribution to study conception and design, data collection, analysis and interpretation of results, and manuscript preparation and reviewed the results and approved the final version of the manuscript.
